# Guide-sheath-assisted bailout technique for intra-procedural single-leaflet device attachment using the PASCAL system: a case report

**DOI:** 10.1093/ehjcr/ytag800

**Published:** 2026-10-24

**Authors:** Masato Nukariya, Yuki Nishimura, Yoko Nakaoka, Kazuya Kawai, Hiroki Sugane

**Affiliations:** Department of Cardiology, Chikamori Hospital, 1-1-16 Okawasuji, Kochi 780-0052, Japan; Department of Cardiology, Chikamori Hospital, 1-1-16 Okawasuji, Kochi 780-0052, Japan; Department of Cardiology, Chikamori Hospital, 1-1-16 Okawasuji, Kochi 780-0052, Japan; Department of Cardiology, Chikamori Hospital, 1-1-16 Okawasuji, Kochi 780-0052, Japan; Department of Cardiology, Chikamori Hospital, 1-1-16 Okawasuji, Kochi 780-0052, Japan

**Keywords:** Case report, Transcatheter edge-to-edge repair, PASCAL system, Single-leaflet device attachment, Bailout technique, Mitral regurgitation

## Abstract

**Background:**

Single-leaflet device attachment (SLDA) is a rare but serious complication of mitral valve transcatheter edge-to-edge repair (MTEER). When SLDA occurs after full device release, management options are limited. In contrast, intra-procedural SLDA recognized before full implant release may allow a specific bailout strategy.

**Case summary:**

A 77-year-old man with severe degenerative mitral regurgitation (MR) underwent MTEER using the PASCAL Ace system because of high surgical risk. Initial device implantation reduced MR from severe to mild. However, after removal of both clasp lines during the release process, SLDA occurred at the posterior leaflet, resulting in recurrent severe MR. Although standard retrieval was no longer feasible, the implant remained connected to the steerable catheter. Under fluoroscopic and transoesophageal echocardiographic guidance, a guide sheath was advanced coaxially towards the implant. Controlled insertion of the guide sheath between the clasp and paddle, followed by gradual paddle opening, enabled leaflet disengagement and successful device retrieval without leaflet injury. A second implantation achieved a sufficient reduction in mitral regurgitation.

**Discussion:**

This case demonstrates a novel bailout technique for intra-procedural SLDA. Successful retrieval requires precise coaxial device alignment, careful catheter manipulation, continuous monitoring of leaflet tension, and meticulous guidance using fluoroscopy and transoesophageal echocardiography (TEE). Importantly, prompt recognition of SLDA by TEE is a prerequisite for this technique. In conclusion, the guide-sheath-assisted retrieval technique can represent a potential bailout strategy for intra-procedural SLDA before full implant release during MTEER.

Learning points

- Intra-procedural single-leaflet device attachment recognized before complete device release permits a bailout option using a guide sheath.
- Several technical steps, including coaxial sheath alignment, meticulous imaging guidance, and controlled device manipulation, are crucial to perform this bailout technique.

## Introduction

Single-leaflet device attachment (SLDA) is a potentially device-related complication of mitral valve transcatheter edge-to-edge repair (MTEER), with a reported incidence of ∼1%.^1,2^ When SLDA occurs, leaflet coaptation failure frequently leads to recurrent significant mitral regurgitation (MR).^3,4^ Normally, SLDA is identified by a follow-up echocardiography after the procedure. In contrast, intra-procedural SLDA recognized before full implant release may allow specific bailout strategies. We report an intra-procedural SLDA case successfully managed using a novel bailout technique.

## Summary figure

**Figure:**
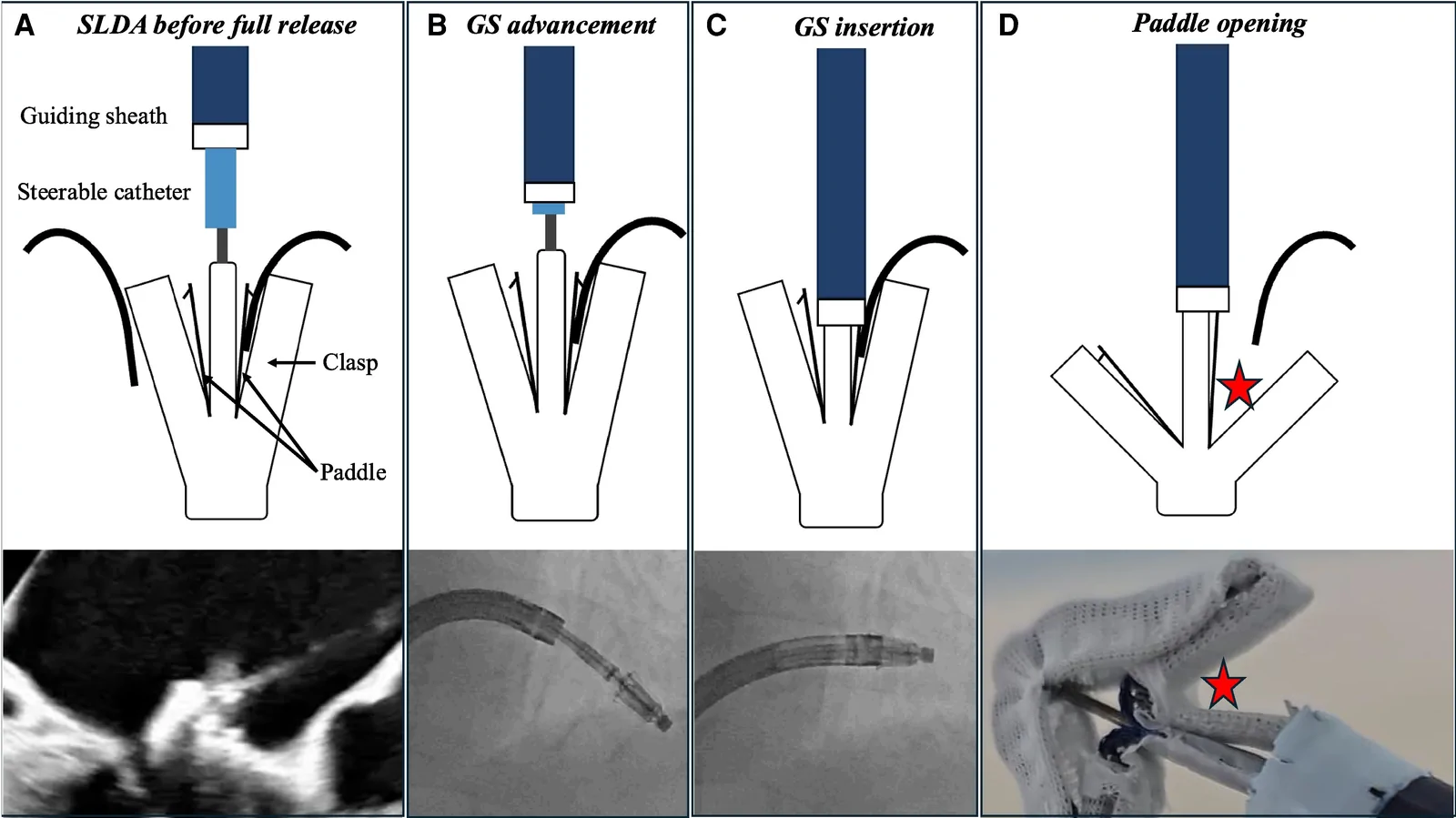
Central Illustration. Overview of the guide-sheath-assisted bailout approach for single-leaflet device attachment. Stepwise schematic overview of the guide-sheath-assisted bailout technique for single-leaflet device attachment. GS, guide sheath; SLDA, single-leaflet device attachment.

## Case presentation

A 77-year-old man was admitted to our hospital with the diagnosis of acute decompensated heart failure. His medical history included long-standing persistent atrial fibrillation, previous cerebral infarction, and chronic obstructive pulmonary disease. His CHA_2_DS_2_-VASc and HAS-BLED scores were 5 and 2, respectively. He had been receiving edoxaban 60 mg/day for stroke prevention. He had no history of prior hospitalization for heart failure or cardiac intervention. His Clinical Frailty Scale score was 4. He presented with dyspnoea at rest and orthopnoea corresponding to New York Heart Association functional class IV. His vital signs were stable except for hypoxaemia, with an oxygen saturation of 92%–94% despite supplemental oxygen at 2–3 L/min. Physical examination revealed significant systolic murmurs, bilateral pulmonary crackles, and bilateral lower-extremity oedema. The electrocardiogram showed atrial fibrillation without significant ST-T change. The chest X-ray showed bilateral pulmonary oedema. The laboratory data showed significantly elevated B-type natriuretic peptide levels of 411 pg/mL. After stabilization of the heart failure with intravenous loop diuretic and vasodilator therapy, further examinations were performed. Before the intervention, he was receiving sacubitril/valsartan 200 mg/day, bisoprolol 2.5 mg/day, spironolactone 25 mg/day, empagliflozin 10 mg/day, and azosemide 30 mg/day for heart failure management.

Transthoracic echocardiography showed severe eccentric MR from the far lateral side of the mitral valve with an effective regurgitant orifice area of 0.43 cm^2^ and regurgitant volume of 64 mL. It also showed an impaired left ventricular ejection fraction of 43%. Transoesophageal echocardiography (TEE) demonstrated severe degenerative MR caused by billowing with prolapse of the anterior commissural segment (*Figure 1*, Supplementary material online, movie  *S1*). The prolapse width and gap were 7.1 and 6.5 mm, respectively. The posterior leaflet length was 8.2 mm. The mean transmitral gradient was 5.1 mmHg, and the mitral valve area was 7.6 cm^2^. Coronary angiography showed no significant coronary artery disease. The Society of Thoracic Surgeons predicted the mortality risk for mitral valve repair of 8.5%, and the EuroSCORE Ⅱ was 7.2%. The Charlson Comorbidity Index was 3. Through shared decision-making with our hospital’s heart team and the patient, MTEER was planned with the PASCAL Ace system (Edwards Lifesciences, Irvine, CA, USA).

**Figure 1:**
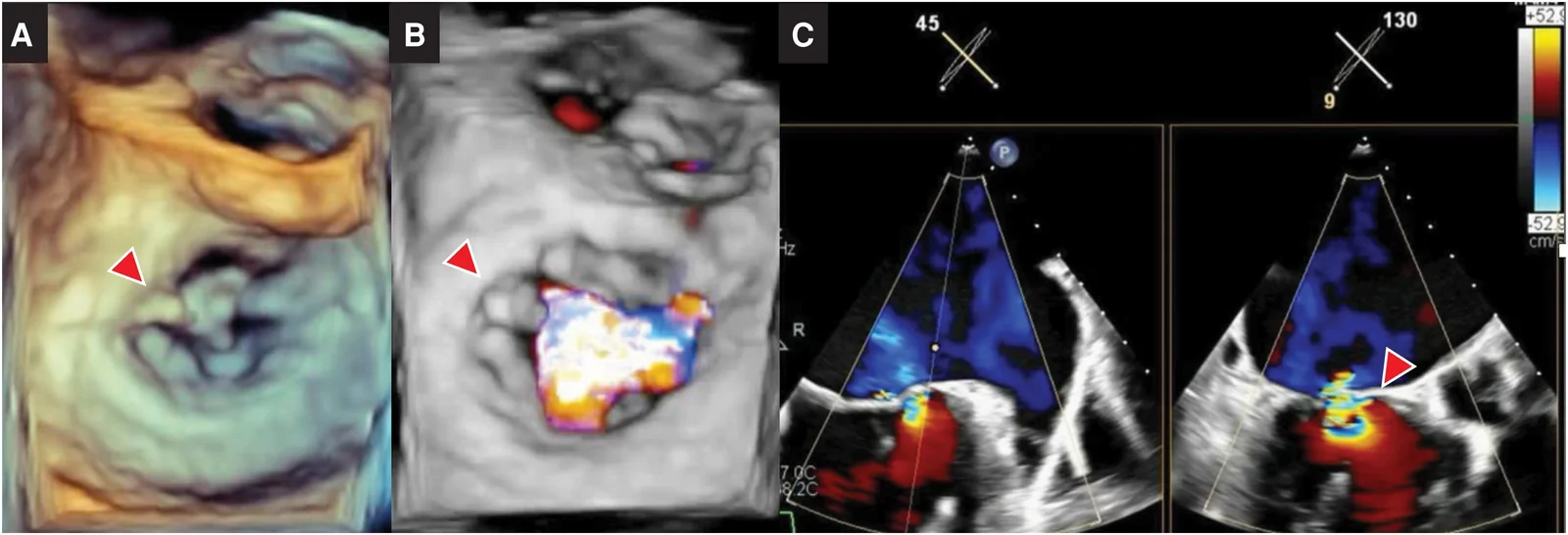
Pre-procedural transoesophageal echocardiography. (*A*) Three-dimensional image without colour; (*B*) three-dimensional image with colour; (*C*) X-plane view at the far lateral side of the mitral valve. Pre-procedural transoesophageal echocardiography demonstrated severe mitral regurgitation caused by significant prolapse at the far lateral side of the mitral valve. Red arrowheads indicate the prolapsed anterior commissure, which was the origin of mitral regurgitation.

The procedure was performed under general anaesthesia and TEE guidance. After the depth of leaflet insertion was confirmed by TEE, the implant was closed. The eccentric jet decreased from severe to mild, and the implant appeared stable (see Supplementary material online, movie  *S2*). Then, the implant-releasing process was initiated. First, the clasp line connected to the paddle that grasped the anterior leaflet was removed. After confirmation of the implant’s stability, the clasp line connected to the paddle that grasped the posterior leaflet was removed. However, after the removal of both clasp lines, intra-procedural SLDA at the posterior leaflet and recurrence of severe MR occurred (*Figure 2*, Supplementary material online, movie  *S3*). At this stage, the implant remained connected to the steerable catheter (SC), and paddle opening or closing remained feasible. However, manipulating the clasps and standard retrieval were impossible. Then, a guiding-sheath-assisted bailout approach was undertaken.

**Figure 2:**
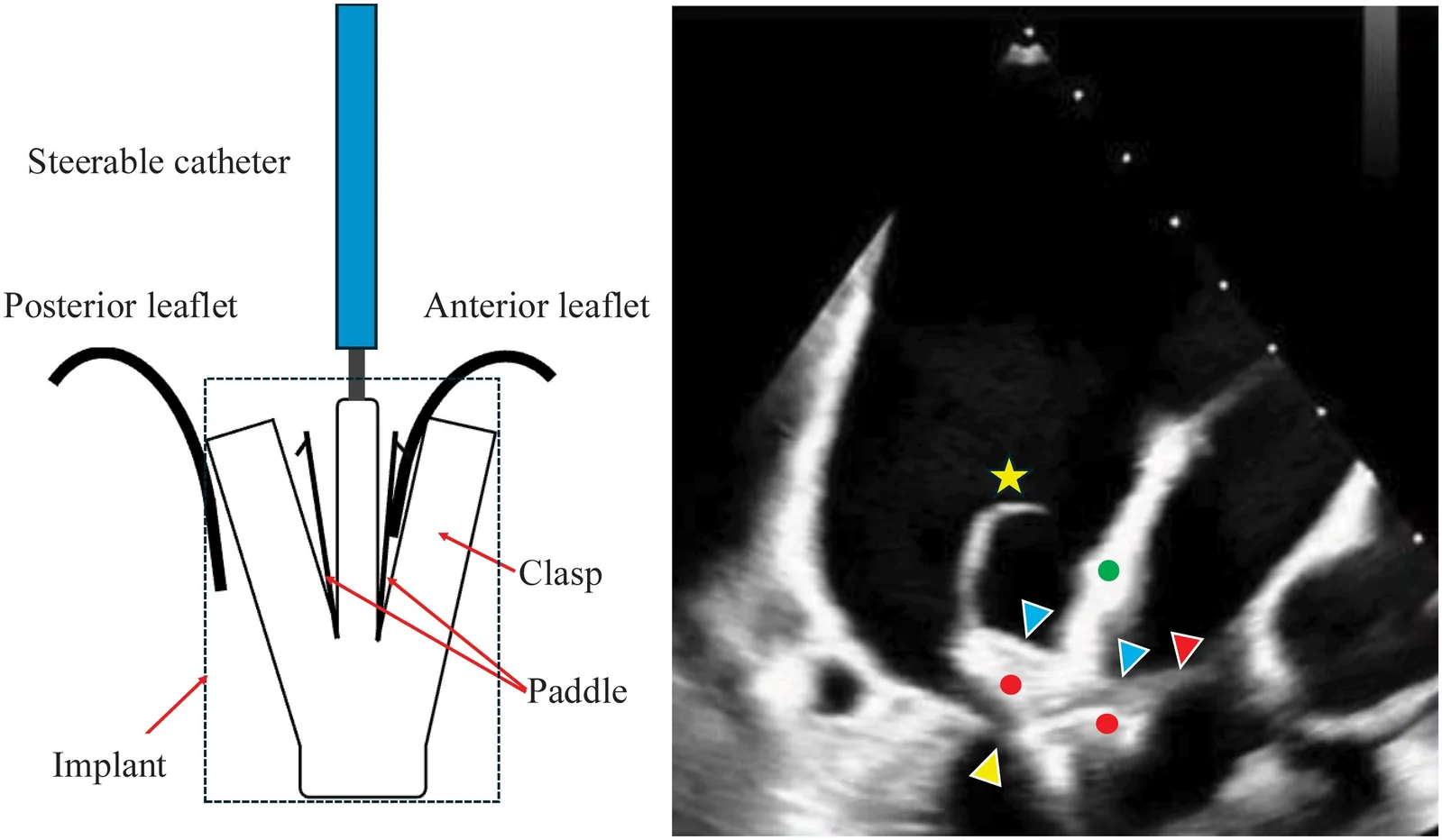
Intra-procedural transoesophageal echocardiography after the occurrence of single-leaflet device attachment. Intra-procedural transoesophageal echocardiography after removal of both clasp lines demonstrated single-leaflet device attachment at the posterior leaflet. ‘Implant’ refers to the entire distal component of the PASCAL system attached to the steerable catheter. The yellow star indicates the posterior clasp line, which was no longer connected to the implant. The red circles indicate the paddles, whereas the blue arrowheads indicate the clasps. The green circle indicates the steerable catheter. The yellow arrowhead indicates the posterior leaflet disengaged from the clasp and paddle, demonstrating single-leaflet device attachment. The red arrowhead indicates the anterior commissure captured by the clasp and paddle. Neither clasp could be further manipulated because both clasp lines had been removed.

First, the guide sheath (GS) was advanced gradually towards the implant while maintaining coaxial alignment with the SC. Coaxial alignment and leaflet tension were controlled through coordinated manipulation of both the GS and the SC (*Figure 3*, Supplementary material online, movie  *S4*). Because this manoeuvre may impose significant tension on the mitral leaflets, careful assessment and control of leaflet tension were performed using TEE.

**Figure 3:**
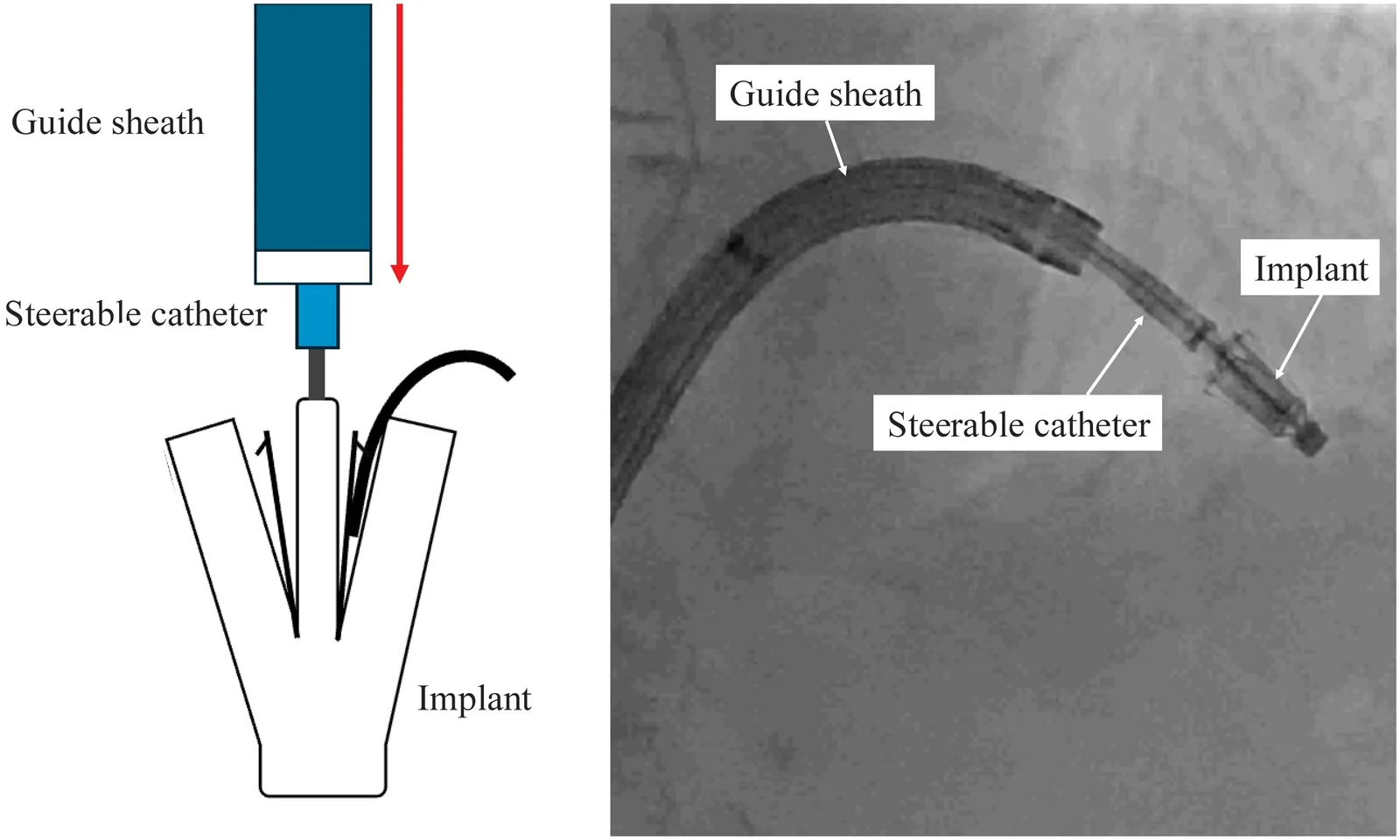
Guide sheath advancement. The guide sheath was advanced towards the implant while maintaining coaxial alignment among the guide sheath, steerable catheter, and implant to minimize tension on the mitral leaflet.

Second, the GS was gently advanced until sufficient insertion depth between the clasp and the paddle was confirmed fluoroscopically, corresponding to approximately one-quarter of the implant length (*Figure 4*, Supplementary material online, movie  *S5*).

**Figure 4:**
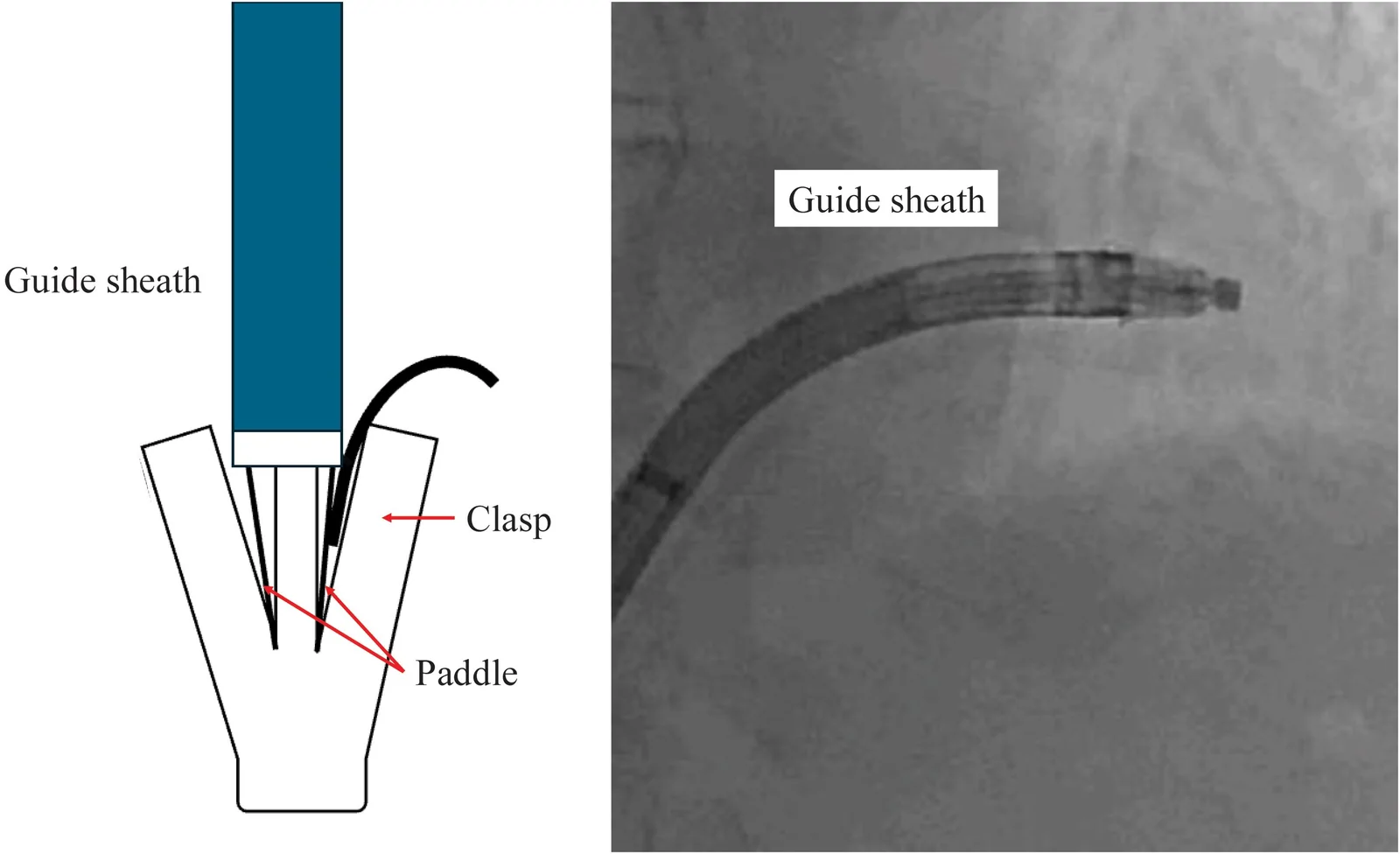
Guide sheath insertion. The guide sheath was gently advanced between the clasp and the paddle to approximately one-quarter of the implant length.

Third, once the guiding sheath was positioned at the target depth, the paddle was gradually opened. Slow paddle opening created controlled separation between the clasp and paddle, allowing leaflet disengagement (*Figure 5*, Supplementary material online, movie  *S6*). Then, the first implant was retrieved, and TEE imaging confirmed no leaflet injury.

**Figure 5:**
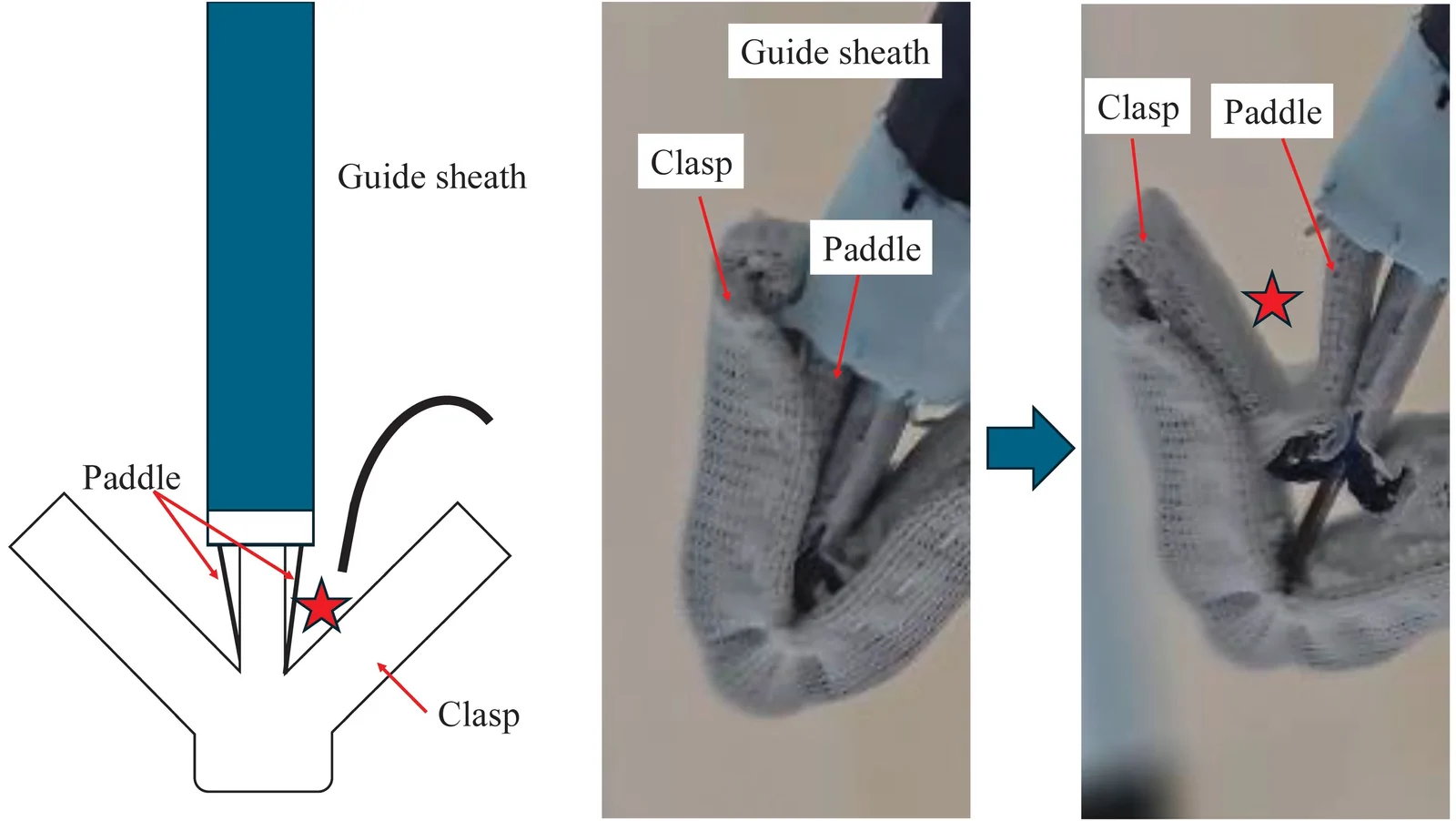
Gradual opening of the paddle. Gradual paddle opening after guide sheath advancement created a gap between the paddle and the clasp (red star) and facilitated leaflet disengagement.

Finally, the second implantation was subsequently performed, and sufficient MR reduction was achieved without further complications (see Supplementary material online, movie  *S7*).

The post-procedural course was uneventful, with improvement in heart failure symptoms; azosemide was discontinued, while the other medications were continued unchanged. The patient was discharged on postoperative Day 5.

## Discussion

This case demonstrates a novel bailout technique for intra-procedural SLDA. Although the applicability of this technique is limited, it may serve as a last-resort bailout option for intra-procedural SLDA. In this case, three important points are highlighted.

First, step-by-step TEE confirmation during the release process and immediate recognition of SLDA are essential. If at least one clasp line remains connected to the device, both clasps remain functionally controllable, allowing leaflet release and system retrieval without this bailout technique. In addition, if the SC is completely removed from the implant, this bailout technique cannot be applied. Therefore, at each step of the release process, device stability and SLDA should be confirmed.

Second, this technique should be applied under meticulous TEE and fluoroscopic guidance. This technique can cause significant tension on the mitral valve leaflets. Excessive tension on the leaflets may cause haemodynamic instability and increase the risk of leaflet injury. Therefore, assessment of the leaflet tension on TEE is crucial.

In addition, to avoid excessive tension on the leaflets, it is important to maintain coaxial alignment among the device components, including the GS, SC, and implant. For this purpose, the alignment of the entire system should be confirmed by fluoroscopic imaging.

Third, the feasibility of this technique has been demonstrated only for MTEER using the PASCAL system. Because the PASCAL implant’s fixation mechanism is shape-memory, once the separation between the clasp and the paddle is achieved, implant retrieval is feasible. In addition, the configuration of the PASCAL, comprising the GS, SC, and implant, enables the coordinated manipulation required for this bailout technique. Theoretically, this technique can be applied in tricuspid transcatheter edge-to-edge repair. However, its feasibility in other situations should be evaluated carefully in the future.

In conclusion, this guide-sheath-assisted bailout technique provides a potential bailout option for intra-procedural SLDA. The technique relies on precise catheter manipulation, controlled coaxial alignment, careful management of leaflet tension, and, most importantly, prompt recognition of SLDA by TEE.

## Lead author biography


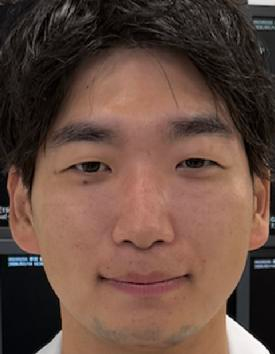


Masato Nukariya, MD, received his medical degree from Tokyo Medical University in 2019 and is currently a cardiologist in the Department of Cardiology at Chikamori Hospital in Japan. His clinical and research interests include structural heart disease, transcatheter aortic valve implantation, transcatheter mitral valve intervention, and complex coronary intervention.

## Supplementary Material

ytag800_Supplementary_Data

## Data Availability

The data underlying this article are available in the article and in its online Supplementary material.

## References

[1] Gupta S, Aggarwal D, Gao M, Bhatia K, Petrovic M, Dominguez AC, et al Single leaflet device attachment after Pascal implantation for transcatheter edge-to-edge repair: a systematic review and meta-analysis. Struct Heart 2025;9:100698. 10.1016/j.shj.2025.100698 PMC12454887 40994668

[2] Bansal A, Puri R, Yun J, Krishnaswamy A, Jilaihawi H, Makkar R, et al Management of complications after valvular interventions. EuroIntervention 2025;21:e390–e410. 10.4244/EIJ-D-24-00066 PMC11995294 40259838

[3] Russo G, Taramasso M. “Mind the Grasp”: preventing single leaflet device attachment and clip embolization in transcatheter edge-to-edge procedure. JACC Case Rep. 2023;9:101747. 10.1016/j.jaccas.2023.101747 PMC9998716 36909268

[4] Hausleiter J, Stocker TJ, Adamo M, Karam N, Swaans MJ, Praz F. Mitral valve transcatheter edge-to-edge repair. EuroIntervention 2023;18:957–976. 10.4244/EIJ-D-22-00725 PMC9869401 36688459

